# Bioartificial Hearts, Assist Devices, and Myocardium: New Developments

**DOI:** 10.1097/TP.0000000000005435

**Published:** 2025-06-24

**Authors:** Patricia Castellanos Vaquero, Anastasiya Rozenbaum, Maria Rocchi, Maziar Arfaee, Paul F. Gründeman, Jolanda Kluin

**Affiliations:** 1 Department of Cardiothoracic Surgery, Erasmus Medical Center, Rotterdam, the Netherlands; 2 Department of Cardiothoracic Surgery, University Medical Center Utrecht, Utrecht, the Netherlands

## Abstract

The rising prevalence of heart failure, global donor heart shortages, and limitations of current assist devices have driven innovation in bioartificial hearts (BAHs) and cardiac constructs. This systematic review aims to give an overview of new developments in BAHs, engineered myocardium, and biohybrid ventricular assist devices research, evaluating their clinical readiness and outcomes while addressing strengths and limitations. Significant variability in study designs and outcomes highlights both advancements and ongoing challenges in this field. Although the development of BAHs and larger cardiac tissue constructs remains in preclinical stages, progress has been achieved in the development of cardiac patches, with 2 approved for clinical use. Several critical challenges continue to hinder the successful clinical translation of bioengineered cardiac solutions. Achieving meaningful myocardial contraction remains a complex task, as well as ensuring adequate vascularization and electrical integration. Biocompatibility limits the progression of bioengineered cardiac constructs toward clinical applications. Innovations in 3-dimensional bioprinting, shape-memory materials, adhesives, microfabrication techniques, and soft and stretchable bioelectronics are driving advancements in this field. However, outcomes regarding hemodynamic performance of BAHs or constructs are marginal at best. Cardiac patches show promising results in preclinical studies, with the paracrine effect of the patches being the most plausible explanation of these results. Importantly, from very little clinical experience thus far, we cannot conclude that cardiac patches have any beneficial effects nor that they are safe. The path toward developing a fully functional BAH or even parts of a functional myocardium appears to be long, complex, and perhaps even unattainable.

## Graphical abstract

**Figure:**
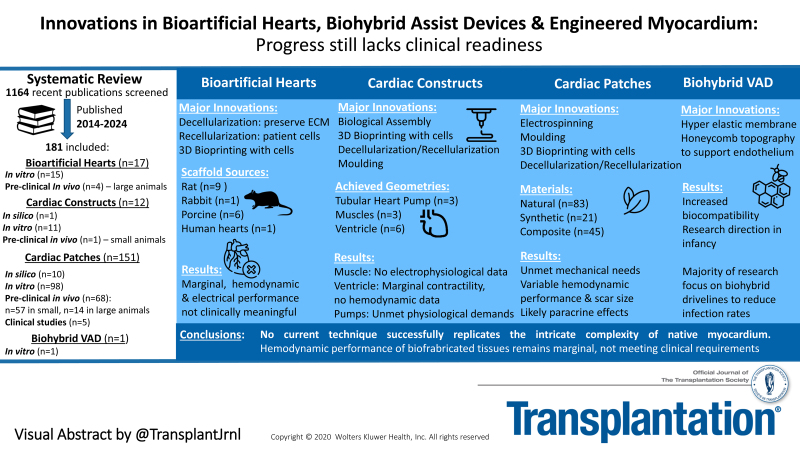

## INTRODUCTION

Heart failure (HF) is a progressive, debilitating condition that affects >64 million people globally.^1^ The incidence of HF is predicted to rise due to an aging population and increasing prevalence of risk factors such as hypertension, diabetes, and coronary artery disease.^1^ Furthermore, the prognosis of HF is poor, with approximately 50% mortality rates within 5 y of diagnosis, with heart transplantation and the implantation of ventricular assist devices (VADs) remaining the gold standard treatments for end-stage disease.^1^ Importantly, from 2010 to 2016, the number of adults on heart transplant waiting lists in the United States surged by 32.3%, reaching 4373 in 2021—the largest increase in the past decade.^2^

During the past 20 y, VADs have seen significant advancements in durability, biocompatibility, and miniaturization, leading to improved patient outcomes.^3^ Nevertheless, infections and thromboembolic events remain the major complications of VAD implantation, with an annual incidence of up to 38.5% and 14.8%, respectively.^4^ The insufficient hemocompatibility of blood-contacting surfaces in current devices remains a critical area for improvement, as the interface between artificial materials and the blood circulation can activate platelets and trigger the coagulation cascade, posing risks of thromboembolic and hemorrhagic events, nonsurgical (gastrointestinal) bleeding complications, and demanding lifelong anticoagulation therapy.^4,5^ The nonphysiological way of propelling blood adds to the poor biocompatibility of current VADs. Thus, the persistent biocompatibility-related complications of current cardiac mechanical support devices, alongside the chronic global shortage of donor organs for transplantation, highlight the pressing need for innovative therapeutic advancements in this field.

One of the alternative approaches that addresses these critical challenges is the development of bioartificial hearts (BAHs), which are engineered constructs designed to replicate the structure and function of natural hearts. By using a combination of biological materials and living cells, researchers aim to create a functional organ that can potentially be used for transplantation or as a temporary support system for patients with advanced stages of HF. These constructs typically involve the use of decellularized scaffolds, which provide a 3-dimensional (3D) framework that retains the extracellular matrix (ECM) properties and the architecture of the native heart tissue, allowing for the repopulation with various cell types, such as cardiomyocytes, endothelial cells, and stem cells, to restore cardiac function.^6,7^

The development of BAHs has evolved significantly during the past decades. The 1990s saw advancements in tissue engineering (TE) principles, including the use of scaffolds and biomaterials, which laid the groundwork for using decellularized matrices for cardiac TE. By the early 2000s, techniques for decellularizing whole hearts were developed, leading to the first reports of decellularized heart scaffolds used in animal models for tissue regeneration.^6^ The 2010s marked a significant leap in the field with innovations in 3D bioprinting and the implantation of complete BAHs in animal models.^6^

Despite significant technological advancements, the fabrication of complete human-sized BAHs encounters major challenges, including sufficient scaffold recellularization, functional integration of cardiac cells, developing bioreactors that facilitate tunable organ-specific maturation, biocompatibility, vascularization, and the maturation of a fully functional, human-sized recellularized heart.^6,7^ Both these critical challenges and recent innovations in biofabrication techniques have led to concurrent developments of cardiac constructs and patches designed to provide mechanical and contractile support to localized infarcted areas of the myocardium while promoting tissue regeneration and functional recovery through the delivery of bioactive factors and cells.^6-9^ Recent advancements in bioengineering have facilitated the fabrication of larger and more complex cardiac constructs, such as whole ventricles.^8-13^ These innovations involve integrating various techniques, including 3D bioprinting, which allows for precise layering of cells and biomaterials to replicate the intricate architecture of cardiac tissue.

This systematic review aims to evaluate and summarize research conducted from 2014 to 2024 on BAHs, cardiac constructs and patches, and hybrid approaches in VADs. Additionally, it seeks to identify the challenges and opportunities related to the advancement of these innovative technologies toward clinical translation.

## MATERIALS AND METHODS

This systematic review was conducted in accordance with the Preferred Reporting Items for Systematic Reviews and Meta-Analyses guidelines.^14^ It focused on state-of-the-art advancements in BAHs, engineered myocardium (including cardiac patches and constructs), and biohybrid VADs.

### Literature Search Strategy

A systematic literature search was conducted in the electronic databases MEDLINE, Embase, and Web of Science to identify articles published between January 2014 and July 11, 2024. Seminal articles before 2014 were included through citation chaining to ensure a thorough and comprehensive overview of the key milestones in the field. Search strategies for the databases are provided in Table 1, illustrating the relevant keywords and Medical Subject Headings terms. The definitions of the terms commonly used in the review can be found in **Supplemental Materials and Methods** (**SDC**, https://links.lww.com/TP/D274; Table 2).

**TABLE 1.** Syntax MEDLINE, Embase, and Web of Science databases

	Database	Search syntax
1	MEDLINE	(((* Bioartificial Organs/ OR * Decellularized Extracellular Matrix/ OR * Bioengineering/ OR * Bioprinting/ OR * Tissue Engineering/ OR * Biocompatible Materials/) AND (* Heart/ OR * Heart Function Tests/ OR * Heart Failure/ OR * Heart Ventricles/ OR * Myocardium/ OR * Heart Transplantation/ OR exp * Heart-Assist Devices/)) OR (((bioartificial* OR bio-artificial* OR decellular* OR acellular* OR bioprint* OR bioengineer* OR bio-engineer* OR tissue-engineer* OR biomaterial* OR bio-material* OR electrospinning*) ADJ6 (heart* OR cardiac* OR myocard* OR lvad OR ventric*)) OR ((engineer* OR artificial*) ADJ6 (heart* OR cardiac* OR myocard* OR cardiovascular* OR cardio-vascular*) ADJ6 (tissue* OR regenerat* OR muscle* OR scaffold* OR extracellular-matri* OR organoid*)) OR ((biohybrid*) ADJ (heart* OR cardiac* OR myocard*)) OR hybrid-heart* OR engineered-heart*).ti. OR (((bioartificial* OR bio-artificial* OR decellular* OR bioprint* OR bioengineer* OR bio-engineer* OR tissue-engineer* OR biomaterial* OR bio-material* OR electrospinning*) AND (heart* OR cardiac* OR myocard* OR lvad OR ventric*))).ti.) NOT (exp * Heart Valves/ OR exp * Transcatheter Aortic Valve Replacement/ OR * Extracorporeal Shockwave Therapy/ OR exp * Percutaneous Coronary Intervention/ OR * Intra-Aortic Balloon Pumping/ OR * Catheters/ OR * Cardiac Catheterization/ OR * Catheterization/ OR * Cardiopulmonary Bypass/ OR * Fontan Procedure/ OR (valve* OR extracorpor* OR extra-corpor* OR paracorpor* OR para-corpor* OR percutane* OR (aort* ADJ3 balloon*) OR IABP OR TandemHeart OR Impella OR ecmo OR catheter* OR bypass OR fontan*).ti.) NOT (news OR congres* OR abstract* OR book* OR chapter* OR dissertation abstract*).pt. AND 2014:2025.(sa_year). AND english.la.
2	Embase	(‘bioartificial heart’/mj OR ‘engineered heart tissue’/mj OR ((‘bioartificial organ’/mj OR decellularization/mj OR bioengineering/mj OR bioprinting/mj/exp OR ‘tissue engineering’/mj OR biomaterial/mj OR electrospinning/mj) AND (heart/mj OR ‘heart tissue’/mj OR ‘heart function’/mj OR ‘heart failure’/mj/exp OR ‘heart ventricle’/mj/exp OR ‘cardiac muscle’/mj OR ‘heart transplantation’/mj/exp OR ‘cardiac patch’/mj OR ‘heart assist device’/mj/exp)) OR (((bioartificial* OR bio-artificial* OR decellular* OR acellular* OR bioprint* OR bioengineer* OR bio-engineer* OR tissue-engineer* OR biomaterial* OR bio-material* OR electrospinning*) NEAR/6 (heart* OR cardiac* OR myocard* OR lvad OR ventric*)) OR ((engineer* OR artificial*) NEAR/6 (heart* OR cardiac* OR myocard* OR cardiovascular* OR cardio-vascular*) NEAR/6 (tissue* OR regenerat* OR muscle* OR scaffold* OR extracellular-matri* OR organoid*)) OR ((biohybrid*) NEXT/1 (heart* OR cardiac* OR myocard*)) OR hybrid-heart* OR engineered-heart*):ti OR (((bioartificial* OR bio-artificial* OR decellulari* OR bioprint* OR bioengineer* OR bio-engineer* OR tissue-engineer* OR biomaterial* OR bio-material* OR electrospinning*) AND (heart* OR cardiac* OR myocard* OR lvad OR ventric*))):ti) NOT (‘heart valve’/exp/mj OR ‘heart valve surgery’/exp/mj OR ‘extracorporeal therapy’/exp/mj OR ‘percutaneous coronary intervention’/exp/mj OR ‘aortic balloon’/mj OR catheter/de OR ‘heart catheterization’/exp/mj OR catheterization/mj OR ‘cardiopulmonary bypass equipment’/exp/mj OR ‘Fontan procedure’/mj OR (valve* OR extracorpor* OR extra-corpor* OR paracorpor* OR para-corpor* OR percutane* OR (aort* NEAR/3 balloon*) OR IABP OR TandemHeart OR Impella OR ecmo OR catheter* OR bypass OR fontan*):ti) NOT ([conference abstract]/lim OR [letter]/lim OR [erratum]/lim) AND [2014-2025]/py AND [english]/lim
3	Web of science	TI=(((((bioartificial* OR bio-artificial* OR decellular* OR acellular* OR bioprint* OR bioengineer* OR bio-engineer* OR tissue-engineer* OR biomaterial* OR bio-material* OR electrospinning*) NEAR/5 (heart* OR cardiac* OR myocard* OR lvad OR ventric*)) OR ((engineer* OR artificial*) NEAR/5 (heart* OR cardiac* OR myocard* OR cardiovascular* OR cardio-vascular*) NEAR/5 (tissue* OR regenerat* OR muscle* OR scaffold* OR extracellular-matri* OR organoid*)) OR ((biohybrid*) NEAR/1 (heart* OR cardiac* OR myocard*)) OR hybrid-heart* OR engineered-heart*) OR (((bioartificial* OR bio-artificial* OR decellulari* OR bioprint* OR bioengineer* OR bio-engineer* OR tissue-engineer* OR biomaterial* OR bio-material* OR electrospinning*) AND (heart* OR cardiac* OR myocard* OR lvad OR ventric*)))) NOT ((valve* OR extracorpor* OR extra-corpor* OR paracorpor* OR para-corpor* OR percutane* OR (aort* NEAR/2 balloon*) OR IABP OR TandemHeart OR Impella OR ecmo OR catheter* OR bypass OR fontan*))) AND DT=(article) AND LA=(english) AND PY=(2014-2025)

**TABLE 2.** Definitions of the terms commonly used in the review

Term	Synonyms found in literature	Definition
Bioartificial hearts	Tissue-engineered hearts, bioengineered hearts, biohybrid hearts	Implantable devices that combine biological tissues and engineered components to replicate the pumping function of the heart and often involve decellularization and recellularization of ECM
Cardiac patches	Engineered myocardium, cardiac (tissue) constructs, cardiac patch constructs, cardiac scaffolds, conductive cell-delivery construct, bioengineered patch, matrices, matrix composites, stem cell sheets, human-engineered heart tissue strips	Engineered tissues designed to repair or replace damaged sections of the myocardium and often incorporate biomaterials and cellular components to promote integration and functional recovery of the heart tissue
Cardiac constructs	Engineered myocardium, cardiac patches (if applicable), artificial muscle filaments, bioartificial heart muscle, engineered heart muscle, artificial heart muscle, heart muscle tissue	They are 3D structures made from biological materials that can mimic the architecture and function of natural cardiac tissue
Biohybrid VADs	Biologically enhanced VAD Biointegrated VAD Hybrid VAD	They are devices that combine mechanical and biological components to support heart function in patients with heart failure. Hybrid VADs are designed to enhance biocompatibility and reduce complications associated with traditional mechanical support systems

3D, 3-dimensional; ECM, extracellular matrix; VAD, ventricular assist device.

### Study Selection and Data Extraction

The final literature search was conducted on July 11, 2024. Identical abstracts were removed automatically using Mendeley (Mendeley Desktop, version 1.19.9, Mendeley Ltd). The remaining duplicates were removed in the Systematic Review Software (Veritas Health Innovation, Melbourne, Australia. Available at www.covidence.org.). Two researchers (A.R. and P.C.V.) independently screened each record by title and abstract and subsequently full-text using Covidence Software (Veritas Health Innovation, Melbourne, Australia).

Relevant data were extracted independently by 2 researchers (A.R. and P.C.V.), with each researcher verifying the other’s data entries. The data collection was organized into 3 main categories: study characteristics, (bio)fabrication techniques, and outcomes. Detailed information on the selection criteria and data extraction can be found in **Supplemental Materials and Methods** (**SDC**, https://links.lww.com/TP/D274, article characteristics).

### Quality of Reporting Assessment

Due to the nonstandardized and nonmonitored nature of most preclinical studies, a standard risk of bias analysis could not be conducted. Instead, we used a custom-designed questionnaire to assess biases related to study design and reporting, adapted from Uiterwijk et al^15^ (Table 3). We reviewed all available information from each article, including **Supplemental Materials and Methods** (**SDC**, https://links.lww.com/TP/D274), references, and appendices. One researcher (A.R.) evaluated the reporting quality, whereas a second researcher (P.C.V.) verified the data entries.

**TABLE 3.** Quality assessment of animal studies

		Yes	No	Unclear
		n/total (%)	n/total (%)	n/total (%)
Animal information				
1	Is the animal species described?	74/74 (100%)	0/74 (0%)	0/74 (0%)
2	Is the strain described?	68/74 (92%)	6/74 (8%)	0/74 (0%)
3	Is the number of animals described per experimental group?	61/74 (82%)	13/74 (18%)	0/74 (0%)
4	Is the sex of the animals described?	54/74 (73%)	13/74 (18%)	7/74 (9%)
5	Is the age of the animals described?	24/71^*a*^ (34%)	47/71^*a*^ (66%)	0/71^*a*^ (0%)
6	Is the weight of the animals described?	29/71^*a*^ (41%)	42/71^*a*^ (59%)	0/71^*a*^ (0%)
**7**	Ethical review permission described?	59/71^*a*^ (83%)	12/71^*a*^ (17%)	0/71^*a*^ (0%)
Study design				
8	Is the duration of the follow-up time of the explanations clear?	62/74 (83%)	0/74 (0%)	12/74 (17%)
9	Was random allocation to the groups clearly described?	32/71^*a*^ (45%)	39/71^*a*^ (55%)	0/71^*a*^ (0%)
10	Was the qualitatively echo assessment performed in blinded fashion?	32/71^*a*^ (45%)	39/71^*a*^ (55%)	0/71^*a*^ (0%)
Adverse events				
11	Are adverse events clearly stated?	26/74 (35%)	45/74 (61%)	3/74 (4%)

a Seventy-one instead of 74 because these parameters were not available in 3 articles.

### Model Maturity and Clinical Readiness

Clinical readiness and model maturity were assessed using an 8-stage model maturity scale^16,17^ to evaluate the progression of cardiac models from initial concept to post-market application. The process begins with conceptualization and progresses through stages such as model proposal, prototyping, and development, refining the model using in silico and in vitro methods. It concludes with model validation, animal studies, clinical outcome evaluation, and post-market research to assess safety, efficacy, and long-term clinical outcomes.^16,17^

### Data Analysis

We performed descriptive statistics to characterize the distribution of the collected variables. Figures were created using MATLAB (MathWorks, Natick, MA; MathWorks [n.d.]. MATLAB; retrieved from https://www.mathworks.com).

## RESULTS

### Literature Search and Screening

**FIGURE 1.**
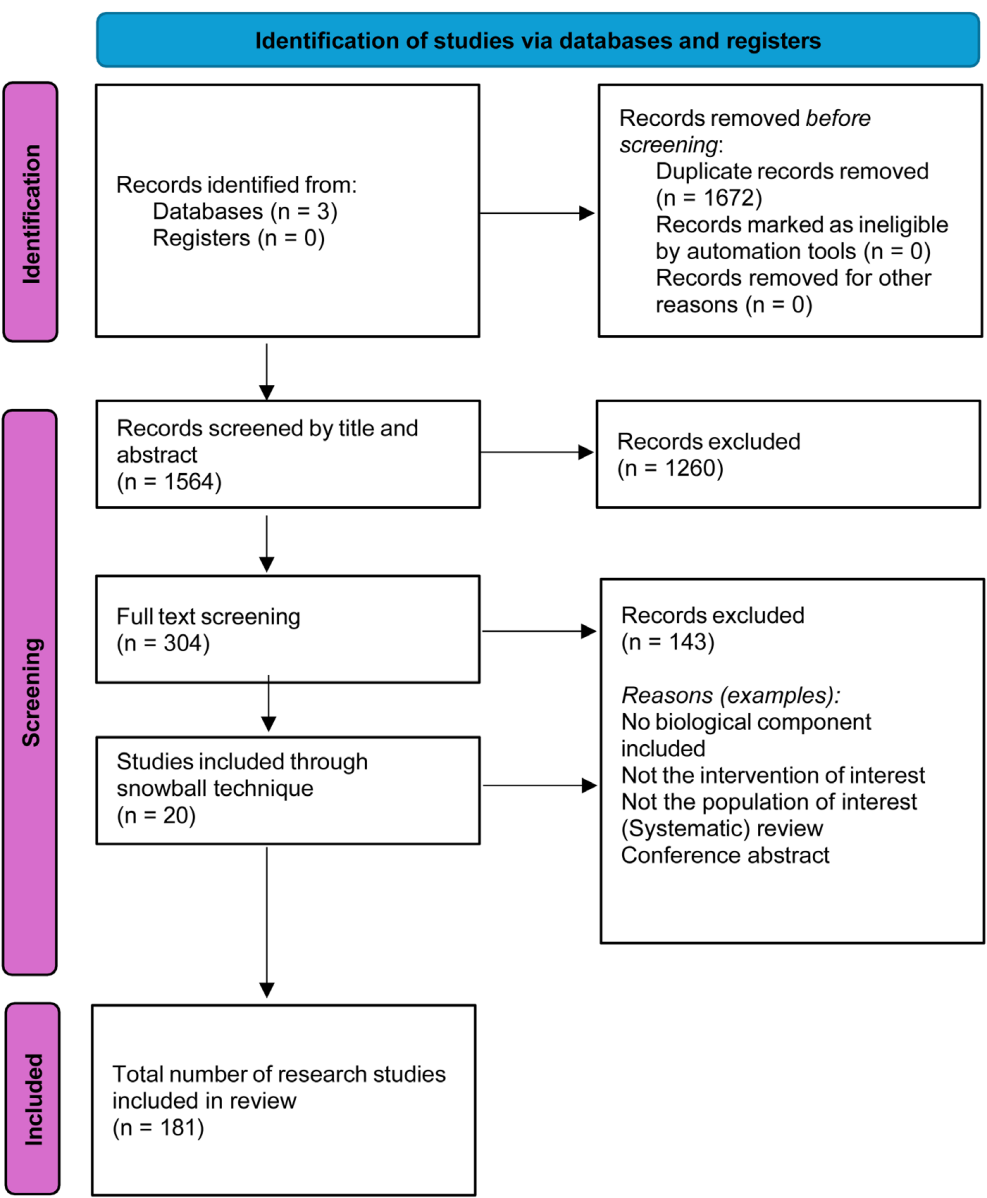
PRISMA search results. PRISMA, Preferred Reporting Items for Systematic Reviews and Meta-Analyses.

**FIGURE 2.**
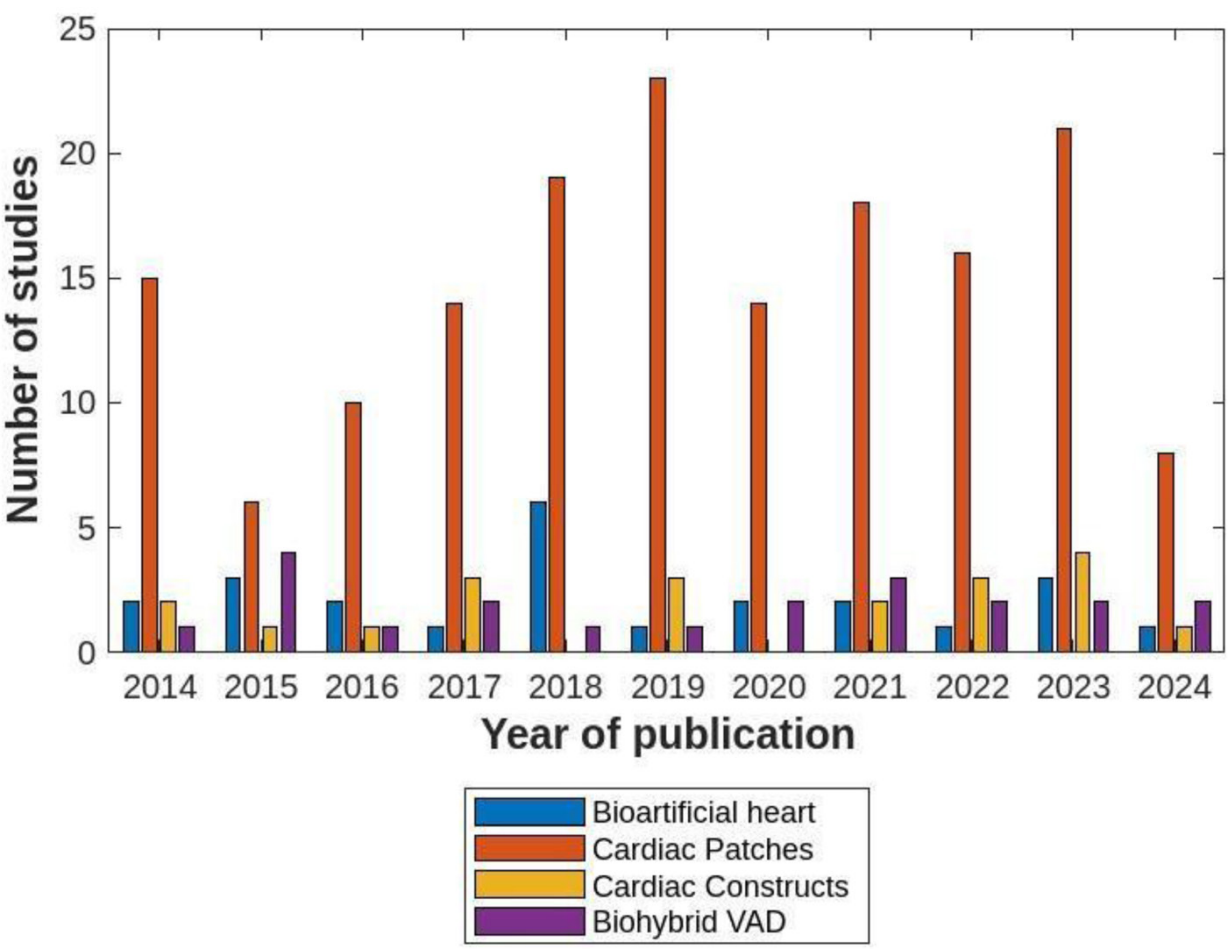
Number of studies published annually (2014–2024) by research focus. VAD, ventricular assist device.

The systematic literature search (July 11, 2024) yielded 1273 publications in Embase and 1963 in the MEDLINE and Web of Science databases. The study selection process is illustrated in the Preferred Reporting Items for Systematic Reviews and Meta-Analyses flow diagram in Figure 1. The number of studies published annually can be found in **Supplemental Materials** (**SDC**, https://links.lww.com/TP/D274; Figure 2). After removing duplicates, 1564 articles were eligible for abstract and title screening. A total of 1260 publications were excluded because of irrelevant interventions or populations, such as TE approaches used for disease modeling or drug screening. The remaining 304 publications were selected for full-text screening, with an additional 20 research publications retrieved from the manual references search through the reference-chaining approach. Finally, we included 181 studies.

### Study Characteristics

The included original research manuscripts covered topics ranging from BAH development (n = 17), cardiac constructs (n = 12), cardiac patches (n = 151), as well as biohybrid VADs (n = 1). One hundred eighty-one studies used a wide range of study designs, including 12 in silico studies, 124 experimental in vitro studies, 74 preclinical in vivo studies, and 5 clinical studies. The animal studies largely focused on small animal studies (n = 59); those with large animal models were sheep, pigs, primates, or goats (n = 16). The follow-up time of the studies ranged from acute (hours) to 6 mo postimplantation.

### Model Maturity: Clinical Readiness

**FIGURE 3.**
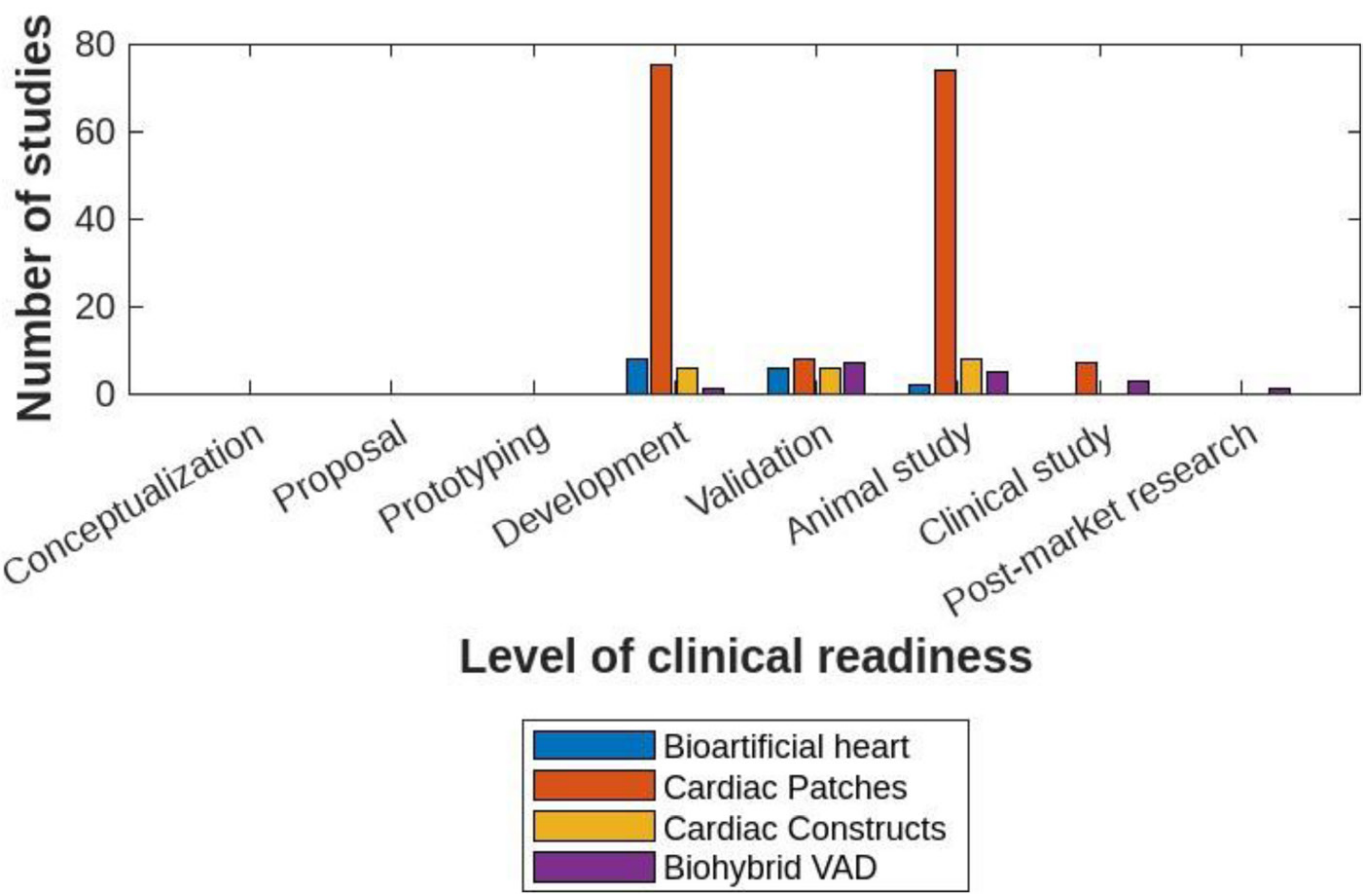
Level of clinical readiness. VAD, ventricular assist device.

Most of the studies were in the model development (n = 81) and animal study (n = 73) phases, with fewer studies reaching the model validation (n = 22) or clinical phase (n = 5; Figure 3). This segmentation provides insight into the maturity of these technologies and their readiness for translation into clinical practice. Notably, only hybrid approaches in VADs and cardiac patches have advanced to clinical studies. The majority of research remains in the in vitro and preclinical in vivo stages, with a substantial focus on cardiac patches.

### Bioartificial Hearts

BAHs have traditionally been constructed using the original matrix of a complete natural heart that has undergone a decellularization process. This process removes the cellular components from donor hearts or tissues, leaving behind a scaffold primarily composed of the ECM that retains the natural architecture.

The first complete BAH fabrication was demonstrated in the study of Ott et al,^6^ when a procured rat heart was decellularized using a perfusion-based technique and recellularized with neonatal rat cardiomyocytes. The authors obtained functional measurements in 5 of 8 constructs. Functional in vitro assessment on day 8 demonstrated contractions on pacing. However, the BAH only achieved about 25% of the neonatal systolic pressure (~15 mm Hg) against an afterload varying between 1 and 60 mm Hg in the Langendorff set-up, although showing enhanced contractile performance using doses of inotropic phenylephrine. Although the study demonstrated contractions in response to pacing, it highlighted the challenge of achieving the physiological contractile force of an adult heart, a critical barrier to clinical translation. Heterotopic transplantation was performed through an end-to-side anastomosis of the donor heart’s ascending aorta and left pulmonary artery to the recipient rat’s abdominal aorta and vena cava, respectively. No contractile performance was reported.

**FIGURE 4.**
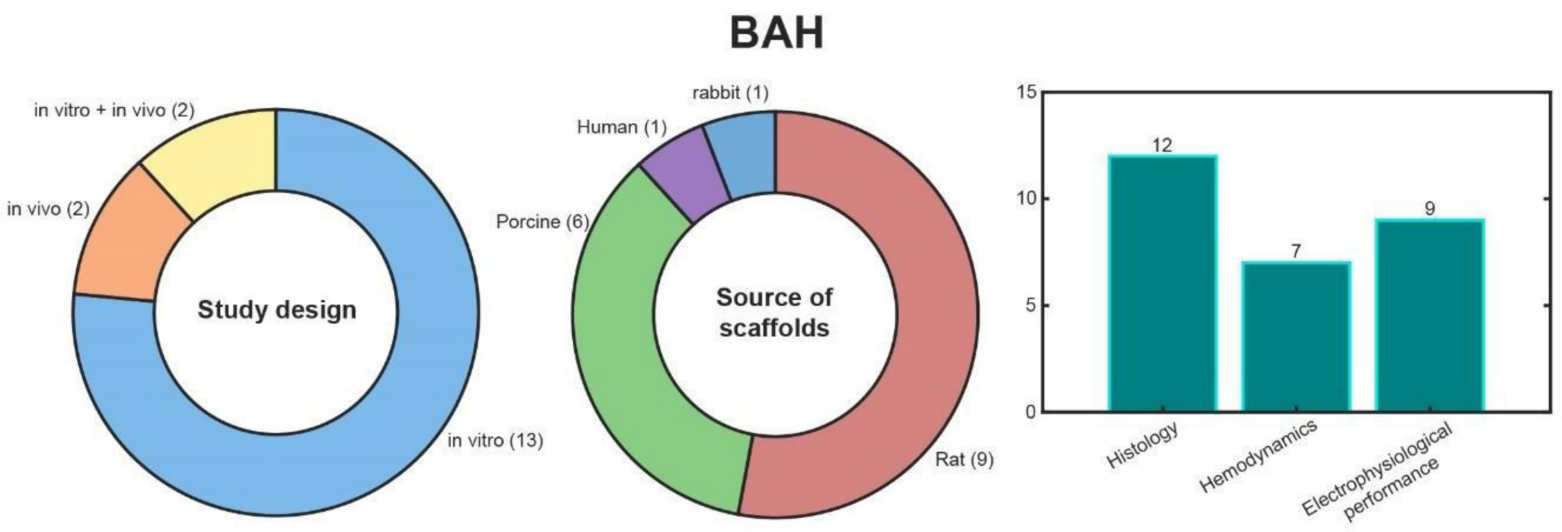
BAH results. BAH, bioartificial heart.

Fifteen studies on BAH fabrication describe in vitro experiments^6,14-27^ and 4 describe preclinical in vivo studies involving heterotopic transplantations in large animal models (Figure 4).^7,26-28^ The studies largely focused on electrophysiological performance (n = 9),^6,14,15,18,19,21-23,25^ histological analysis (n = 12),^7,15,19,24,26-33^ and hemodynamic performance (n = 7; Figure 4).^6,7,14,15,20,22,26^

Rat (n = 9),^6,14,15,17,18,21-23,26^ rabbit (n = 1),^27^ and porcine hearts (n = 6)^7,16,19,20,24,27,28^ have been the primary scaffolds used in BAH development (Figure 4), with recent studies expanding the approach to nontransplantable human hearts (n = 1).^25^ BAH fabrication typically involves chemical decellularization, often using ionic detergents (eg, sodium dodecyl sulfate, n = 11)^6,7,14,15,19,20,23,24,26,27,33^ and nonionic agents, such as Triton X-100 (n = 9).^6,15,18,21,23,24,26,28,30^ According to Ye et al,^24^ sodium dodecyl sulfate demonstrated greater efficiency in decellularization (>90% of DNA removed) compared with trypsin and Triton X-100 (<80% of DNA removed), while also better preserving the microstructure of the ECM for recellularization. Various cell types have been used for recellularization. For example, primary cardiac cells isolated from neonatal rats were cultured within 3D fibrin gels or injected into decellularized scaffolds (n = 5).^15,21,22,24,30^ Mesenchymal stem cells were infused into the aorta or injected into the ventricular wall (n = 3).^23,25,28^ Human embryonic stem cell-derived cardiac progenitor cells, alongside basic fibroblast growth factor, were used to promote cell proliferation and differentiation (n = 1).^18^ Human umbilical vein endothelial cells were perfused into coronary arteries to aid vascular recellularization (n = 2).^19,25^ Three studies used combinations of cardiomyocytes, fibroblasts, and endothelial cells to enhance recellularization (n = 3).^15,27,31^ Two studies reported not using recellularization, instead relying on endogenous regenerative capabilities (n = 2).^7,20^

One of the main challenges of recellularization is ensuring proper cell adhesion and survival within the scaffold. The study of Weymann et al^19^ reported that recellularization was >50%, primarily around injection sites, with significant attrition in distal regions. A live/death assay showed preserved viability of the reseeded neonatal cardiac cells. The study of Tao et al^14^ confirmed the presence of a large number of cells that were distributed throughout the graft; but of the cells injected, approximately 43.5% were washed away within 2 h after each injection. Compared with the natural rat hearts (1.16 g), the recellularized hearts (0.42 g) were much lighter, constituting 36% of the mass of an adult rat heart. Bioreactors were used in 4 studies to provide mechanical and electrical stimuli to enhance cell maturation and integration.^6,17,19,20^

All 15 in vitro studies that assessed electrical and/or hemodynamic performance reported only marginal performance.^6,14-27^ Rajabi et al^18^ revealed that BAHs exhibited erratic and nonuniform electrophysiological activity with a beating frequency of approximately 46 beats per minute (bpm) and field potential amplitudes ranged from 110 to 140 μV. In a healthy human heart, action potential typically causes voltage changes of 70–100 mV during depolarization, with rapid electrical conduction across the myocardium.^34^ Weymann et al^19^ found that after 10 d of bioreactor perfusion after cell seeding, the BAH demonstrated discrete foci with electric voltage undulations, although without any hemodynamic performance mentioned. The creation of sufficiently thick cardiac sheets is limited by the inability to create the geometry necessary to support the high oxygen and energy demands of cardiomyocytes at a thickness >100 mm – the oxygen/nutrition “diffusion barrier.”^22^ None of the in vitro studies have achieved clinically meaningful hemodynamic performance. Yasui et al^15^ reported the highest intraventricular pressure of still only 0.75 mm Hg, a value far below the threshold needed for clinical relevance.

Four studies involved heterotopic transplantations in large animal models (porcine and bovine)^7,28^ and small animal models (rabbit and rats).^26,27^ The first in vivo study, conducted by Robertson et al^26^ investigated the effect of reendothelialization on clotting. Compared with the acellular control, the reendothelialized BAH showed reduced thrombogenesis. However, heterotopic transplantation of either acellular or reendothelialized heart scaffolds did not lead to significant staining of smooth muscle actin, vimentin, or calretinin, which are common markers for smooth muscle cells, fibroblasts, and mesothelium, respectively. No hemodynamic data were reported after transplantation. A second in vivo study by Kitahara et al^28^ focused on the heterotopic transplantation of a decellularized porcine heart, recellularized with mesenchymal stem cells in a porcine model. The scaffold’s integrity was adequate to withstand surgical procedures and blood pressure. Angiography revealed good perfusion of the right and left coronary arteries, although by day 3, thrombus formation occluded the coronary arteries. Histological analysis showed significant inflammatory cell infiltration in both atria and migration into the ventricular muscle. This study demonstrated that residual DNA in decellularized ECM scaffolds can provoke an immune response in the recipient. Unfortunately, data on hemodynamic performance are not available, suggesting that the heart did not show any electrical or hemodynamic performance.

In a third in vivo study by Taylor et al,^7^ both an acute heterotopic transplantation of a decellularized porcine heart in a porcine model and a chronic heterotopic transplantation in a bovine model were performed. The hearts were not recellularized, as the researchers aimed to harness the recipient’s endogenous repair and regenerative capabilities for BAH maturation. The acute model, designed to test the scaffold’s ability to maintain vascular connections for 4–6 h, revealed that although blood flow was not physiologically normal, recipient blood cells were found to be circulating through the vessels. Endothelial cells from the recipient completely populated the decellularized heart, as evidenced by CD31 and von Willebrand factor staining. In the chronic model (60 d), it appeared that by 12 d, all vessels were occluded in the transplanted decellularized heart. Although not specifically reported, these hearts are expected not to show any hemodynamic performance.

Finally, the study by Hochman-Mendez et al^27^ investigated the perfusability and function of a decellularized rabbit heart with and without recellularization with human-induced pluripotent stem (iPS) cell-derived endothelial cells, cardiomyocytes, and other cardiac cell types in a femoral artery bed of an adult pig. No clinically meaningful results could be reported because of the immediate clotting of the scaffold, both at the macro- and microscopic levels.

Bridging the gap between preclinical studies and future clinical trials for BAHs requires meeting critical cardiac performance standards, such as adequate contractility, functional vascularization, and physiological responsiveness under varying conditions, including rest and exercise. Furthermore, successful translation to the clinic will require addressing challenges in biocompatibility, including minimizing immune rejection and inflammation, which are particularly challenging when using xenogeneic materials, but are important to ensure safety and efficacy of BAHs in real-world clinical settings.

### Cardiac Constructs

**FIGURE 5.**
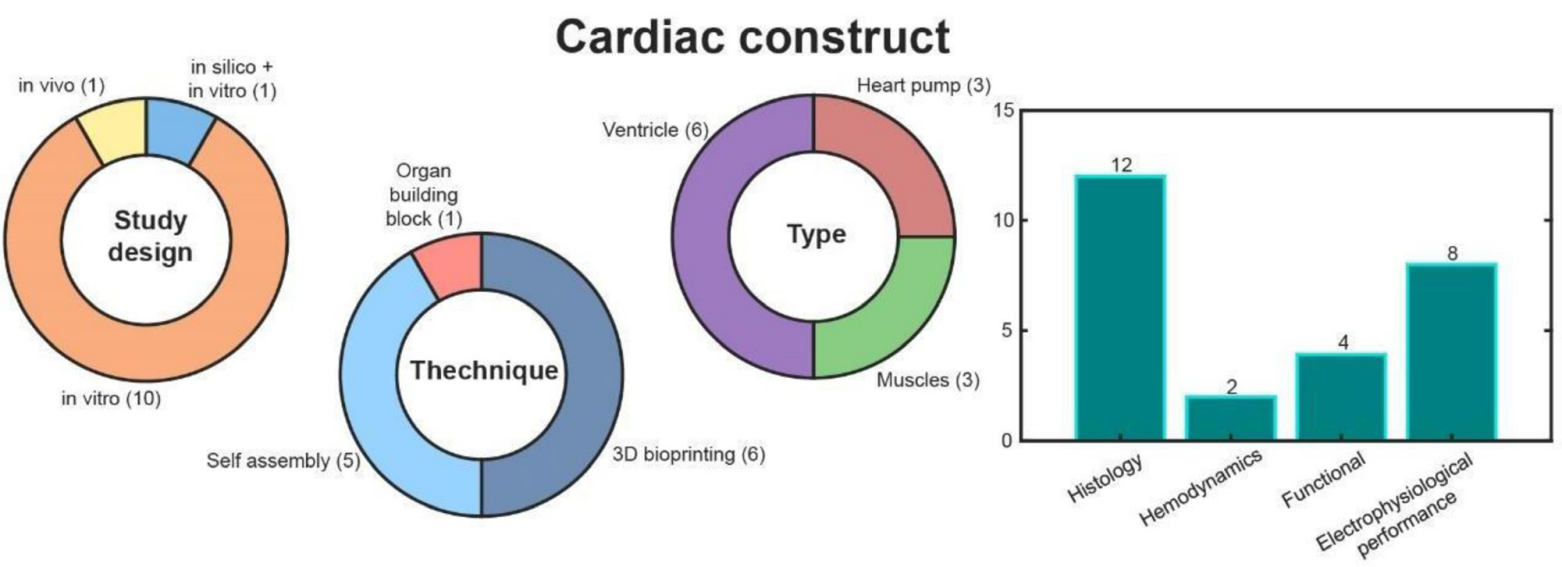
Cardiac construct results.

Cardiac constructs (n = 12)^8-13,35-40^ are engineered myocardial structures designed to replicate the architecture and function of natural cardiac tissue. Studies on cardiac constructs focus on in silico methods (n = 1),^8^ in vitro experiments (n = 11),^8-13,36-40^ and in vivo experiments (n = 1),^35^ with no clinical studies reported (Figure 5).

A range of different geometries of the constructs has been reported: cardiac construct replicating cardiac muscles (n = 3),^35-37^ the entire geometry of the ventricle (n = 6),^8-13^ and a very small TE tubular heart pump (n = 3).^38-40^

Among the 3 studies centered on developing cardiac muscle constructs, 3 TE strategies were used: biological assembly (n = 1),^35^ which involves entrapment of cells in hydrogels; decellularization/recellularization (n = 1),^37^ which entails seeding cells onto a preserved ECM; and 3D bioprinting (n = 1),^36^ which encompasses cell seeding on scaffolds as well as the creation of cell sheets through the serial stacking of monolayer cultures aiming to form contractile tissues. Two studies were performed in vitro,^36,37^ and 1 in a small animal model.^35^

The primary challenge with decellularization/recellularization methods is ensuring enough cell load, as it is crucial to populate the acellular scaffold with sufficient viable cells to achieve functional outcomes. Patel et al^37^ showed a maximum of only 66 ± 23 cells per 1.33 mm² compared with the 825 cells per 1.3 mm^2^ reported in the native tissue. The twitching force reached a maximum of 388.3 μN, which is negligible when compared with that of mammalian tissue, typically ranging from 25 to 44 mN.

Biological assembly techniques offer the advantage of incorporating a high number of cells into the construct, potentially enhancing tissue functionality.^10^ However, a significant challenge remains in promoting vascularization within the structure, which is essential for nutrient and oxygen supply. Jebran et al^35^ showed that only the rat engineered heart muscle grafts with maximal vessel density (425 vessels/mm^2^) showed muscle formation, demonstrating the importance of functional vascularization in providing the required metabolite and oxygen supply. However, the vessel density appears inadequate for cell maturation, as data indicated a drop of 6 ± 4% in Luc-signal intensity after 28 d postsurgery from the baseline luminescence of around 10⁹ p/s/cm²/sr, suggesting a loss of implanted cardiomyocytes.

Finally, 3D bioprinting exhibits a higher potential in the development of more complex architectures with intended spatial arrangements of cells and materials, more closely mimicking natural tissue structures. Yet, 3D bioprinting techniques demonstrated similar challenges to biological assembly constructs. Limitations of 3D bioprinting include a lack of repeatability, construct stability, and cell viability due to insufficient vascularization.^36^

Four studies have examined the mechanical properties of the constructs.^8,35-37^ Tensile testing of the constructs revealed distinct Young’s moduli, recognized as the standard measurement of mechanical performance in relation to material stiffness. In the study by Jebran et al,^35^ a maximum of 46 ± 4 kPa was measured in the engineered mesenchymal tissue and Cui et al^36^ could match the Young’s modulus of the native myocardium during diastole (~10–50 kPa) with the decellularized ECM fiber-reinforced hydrogels composed of 5% gelatin methacrylate and 10% polyethylene glycol diacrylate.

No information is reported on the electrophysiological performance of the developed cardiac muscle constructs. Only limited information was reported regarding the contraction force of the construct in the study by Patel et al.^37^ The twitching force reached a maximum of 388 μN, which is negligible compared with that of mammalian tissue, typically ranging from 25 to 44 mN.

In the field of complete ventricular construct development, research has been conducted either in silico (n = 1)^8^ or in vitro (n = 6).^8-13^ All studies focused on developing a 3D-printed left ventricular (LV) model with dimensions under 3 cm.^8-13^ The main focus of the studies is on the viability of the cells of the ventricular constructs, and not on mechanical performance.

Cetnar et al^8^ found that a physiological flow in the bioreactor enhanced cellular maturation and function. Compared with the static setup, enhanced cellular maturation and function were identified in the dynamic environment for different geometries and flow ranges imposed by the bioreactor. Lee et al^9^ developed a method to print collagen structures to create a porous microstructure that facilitates rapid cellular infiltration and microvascularization. The method allowed for the development of vessels ~100 μm in diameter, far from the 8–10 μm in diameter of the capillaries.^34^ No information is given on the cellular load of the constructs. However, beat rate analysis shows that Lee et al^9^ bioprinted ventricles exhibited a spontaneous rate of about 0.5 Hz (30 bpm), lower than the typical human resting heart rate of 1 to 1.67 Hz (60–100 bpm). Although able to respond to electrical pacing at 1 and 2 Hz (60 and 120 bpm), these constructs still do not match the physiological pacing capabilities of native cardiac tissue, which can adapt to higher rates during physical activity or stress.^34^ The constructs displayed a maximum chamber volume reduction of about 5% during peak systole when tested without afterload, compared with the 40%–50% typical in native hearts having afterload, indicating marginal contractile efficiency.^9,34^

To improve vascularization and increase the cellular load, Skylar-Scott et al^12^ used a novel biomanufacturing technique to assemble hundreds of thousands of organ building blocks into living matrices, achieving a cellular density of approximately 10^8^ cells/mL. These organ-building blocks were derived from patient-specific iPS cells, forming organoids that could mimic the structure and function of the desired tissue. The constructs exhibited spontaneous contractions. However, the deformation during contraction was only 1% of their resting length, significantly lower than the 20% strain typically observed in adult cardiac tissue.^12^

To improve structure integrity of the 3D bioprinted constructs, 2 different approaches were proposed. Patel et al^10,13^ proposed a molding technique. They developed molds both for an open^10^ and closed^13^ ventricle where a solution of chitosan was poured and frozen to create ventricular scaffolds. Those were later cellularized and tested in terms of cell retention, biopotential output, and structural integrity. Around 43% of cells died after the cellularization process.^10^ Despite this, Patel et al^10,13^ reported an average amplitude of 1.62 mV in their bioengineered open ventricle model, whereas the bioengineered complete ventricle achieved a consistent amplitude of 1.02 mV. This aligns with the reference ranges for rat (0.495–0.775 mV) and human (0.1–0.5 mV) electrocardiogram outputs, with the second model more closely matching physiological relevance.

Finally, Hwang et al^11^ proposed a “LEGO”-like structure where simple building block shapes can be assembled via interlocking parts to create various complex structures. The structures were assembled to replicate the myocardial fiber orientations (both for the helical and circumferential layers). In terms of contractile forces, Lee et al^11^ reported 2250 μN for parallel assemblies and 1800 μN for twisted assemblies, compared with 1500 μN for individual modules. Despite these improvements, they remain far below the native heart muscle’s typical range of 20–45 mN, emphasizing ongoing challenges in achieving native-like mechanical properties in engineered tissues.^13^

Three in vitro studies have centered on the development of small scaffold-free tubular cardiac constructs intended to function as TE heart pumps (n = 3).^38-40^ These studies primarily investigated the contraction function and the effects of electrical stimulation on the constructs. Kupfer et al^39^ advanced this field by developing a photo-cross linkable bioink formulation for 3D printing a functional human muscle pump. Their findings revealed spontaneous electrical activity across 56% of the pump’s surface, with an average ejection fraction (EF) of 0.7% and a maximum EF of 6.5%.^41^ Although these results demonstrate the feasibility of creating a heart pump, it is important to recognize that the pumps are very small, and their performance still falls considerably behind the physiological parameters observed in native heart tissue.

### Cardiac Patches

Cardiac patches are bioengineered constructs designed to mechanically reinforce weakened areas of the ventricular wall, typically after myocardial infarction. Beyond mechanical support and possibly active contribution to contractile performance of a localized area, bioactive cardiac patches aim to promote endogenous tissue regeneration and repair, modulate inflammation, provide localized delivery of therapeutic agents, and aid in the restoration of electrical coupling within infarcted regions, therefore potentially contributing to improved overall cardiac function and recovery.

**FIGURE 6.**
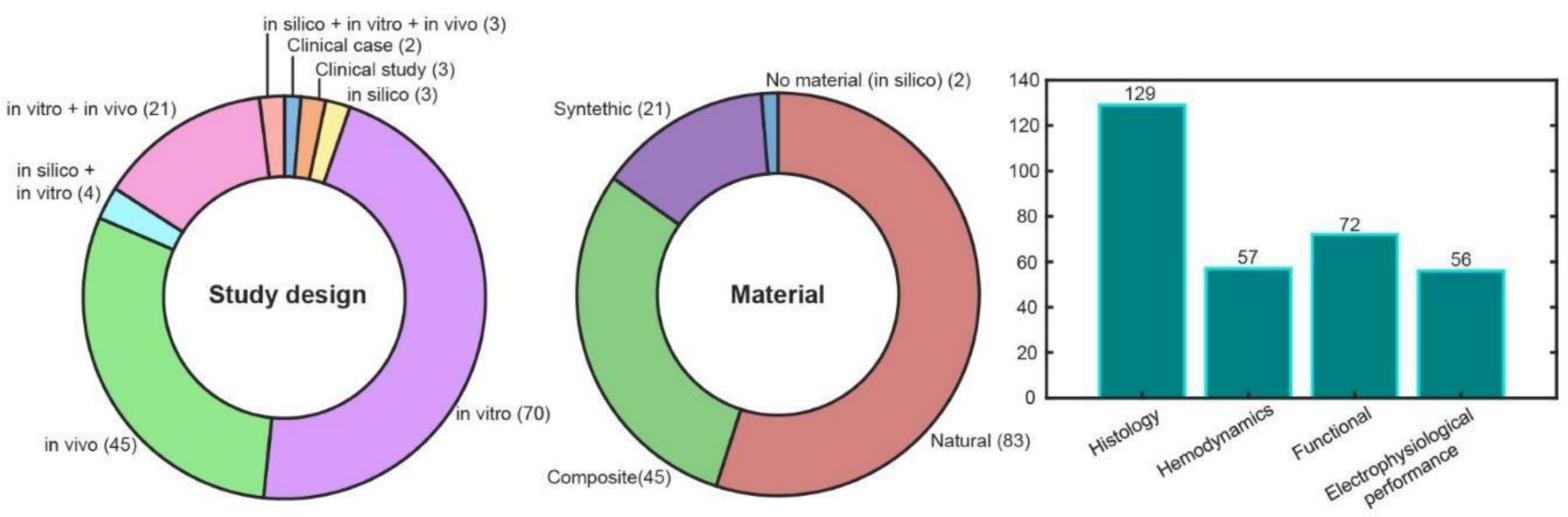
Cardiac patch results.

A total of 151^34,38-188^ studies on cardiac patch fabrication used a variety of study designs, including in silico approaches (n = 10),^42-51^ in vitro methods (n = 98),^43,44,46,49-143^ and preclinical in vivo studies (n = 68).^50-52,55,56,58-64,69,78-80,87,113,117,119,120,130,139,140,144-188^ Of those, 14 preclinical studies were conducted in large animal models^52,60,117,144,145,159,160,169,171,177-179,185,186^ and 57 in small animal models.^50,51,55,56,58,59,61,63,64,69,71,78-80,85,87,105,113,119,120,130,139,140,146-158,161-168,170,172-176,180-187^ Finally, 5 clinical studies were found (Figure 6).^189-193^ Notably, the CorMatrix CorPatch,^162,191^ derived from porcine small intestine submucosa, and PeriCord^189,194^ obtained from allogeneic human pericardium, have received clinical approval from the Food and Drug Administration and the Spanish Agency of Medicines and Medical Devices, respectively, for use in patients with myocardial infarction.

A variety of materials have been used for the fabrication of cardiac patches, including natural (n = 83),^27,43,44,49,52,53,56,57,63-66,70,71,81,82,84,86,87,90-93,99,103,109-111,114,115,117,119,121-124,126,128,129,131-133,136,140-142,145,146,148,150-154,156-163,166-173,175,177,179,181-183,187-189,191-194^ synthetic (n = 21),^51,73,74,77,79,83,85,89,94,95,97,98,101,102,107,127,130,134,138,155,186^ and composite formulations (n = 45).^46,47,50,54,55,58-62,67-69,72,75,76,78,80,88,97,100,104-106,108,113,115,116,118,120,125,137,139,143,144,147,149,164,165,174,176,178,180,184,185^ Natural materials included proteins (eg, fibrin, collagen, gelatin; n = 46),^44,49,53,56,57,63,66,71,81,82,84,86,87,91,93,109,110,112,117,119,121,123,124,128,129,131,141,142,145,148,150,151,154,157,161,166,167,169,170,173,177,182,187,188^ polysaccharides (eg, hyaluronic acid, chitosan, alginate; n = 9),^70,82,87,109,111,117,136,141,154^ as well as decellularized ECM from various sources (n = 24),^49,58,70,81,84,91,99,115,121,126,128,135,136,142,156,159,160,162,167,168,176,183,187,191^ offer superior biocompatibility and the ability to closely mimic native tissue architecture and function.^56,81,151^ However, these cardiac patches often fall short in mimicking the mechanical properties of the cardiac tissue.^56,81,151^

Synthetic materials are widely used in cardiac patches due to their controllable mechanical properties. Key materials include polycaprolactone (n = 1),^107^ polylactic acid (n = 1),^97^ as well as polyurethane (n = 6),^73,89,97,101,102,127^ polyvinyl alcohol (n = 3),^88,130,186^ and polylactic-co-glycolic acid (n = 1).^134^ They offer tunable mechanical properties and degradation rates, allowing for the customization of scaffolds to meet the specific needs of cardiac tissue. However, they can be less biocompatible compared with natural materials and may require surface modification to enhance cell adhesion and bioactivity.

Composite materials that integrate natural and synthetic components synergistically use the biological functionality of natural materials and the superior mechanical properties of synthetic ones. For example, collagen and polycaprolactone blends (n = 4)^50,72,96,165^ have been used to improve mechanical strength while maintaining biocompatibility. Composite formulations allow for the engineering of scaffolds that can both withstand the mechanical stresses of the cardiac motion and promote cell attachment and tissue regeneration by, among others, implementing porous structures.

The primary methodologies used to fabricate synthetic or composite cardiac patches include molding,^69,72,87,160,185,186^ electrospinning,^47,60,65,76,84,88,90,99,100,107,110,144,155,157^ and 3D (bio)printing,^59,82,91,109,137,140,154,174,184,195^ each offering different benefits and limitations. Electrospun fibrous film patches, commonly fabricated from materials such as polycaprolactone, polyurethane, and gelatin, demonstrate good elasticity and a fibrous, biomimetic architecture that aims to replicate the mechanical and structural properties of native ECM, enhancing cell adhesion and proliferation.^47,155^ Nevertheless, such a technique is limited in the geometries that can be created. Molding and 3D (bio)printing techniques can create more complex architectures. Similar to what was described in cardiac constructs, the main limitations include spatial resolution, which represents the ability of the material to be deposited along the x- and y-axes in a predefined way, poor vascularization of the patches, and significant challenges for scaling up for clinical applications. Recent advances in microfabrication techniques, such as soft lithography (n = 1)^107^ and ion etching (n = 5),^51,99,104,151,155^ have enabled the fabrication of microstructures within molds, modifying their topography to include features such as microgrooves, micropatterns, and complex microneedle-negative designs to improve cell adhesion and vascularization.^175,176^

To analyze the performance of a cardiac patch, studies have focused on functional properties (n = 72),^44,46,47,50,51,64,67,72-76,80,83-86,88,89,91-94,96-98,100-106,108-113,116-118,120,122,126,127,129-131,134-139,141,153,155,160,164-167,169-171,180,184,185,192,194,196^ histological parameters (n = 129),^43,46,49-51,53-71,73-93,95,96,101-121,123-126,128-157,159-167,169-185,189^ hemodynamics (n=

57),^36,43,47,52,53,55-58,60,61,63,79,80,99,113,117,119,120,139,140,144,145,147-149,152,154,156,158,162-175,177-179,181,182,184,186,187,189,191-194^ and electrophysiological performance (n = 56).^43,45,51-58,61,65-67,69,71,73-78,80,82,85,86,92,104,114-116,119,121,124,125,129,131,134,137,138,141,143,144,147,149,152,153,155,158,161,163,168,169,178,180,188^

Histological analyses are commonly performed to analyze the cell viability of the cardiac patch, as well as its vascularization. The viability of cells within cardiac patches varies depending on factors such as the type of cells used, the scaffold material, the in vitro or in vivo environment, and the duration of the study, but typically ranges from 70% to 90%.^51,59,60,64,67,71,189^ Different solutions were found to improve the cell viability of a cardiac patch. The study by Bagdadi et al^131^ demonstrated that the incorporation of porous structures into poly(3-hydroxyoctanoate) patches resulted in a 2.5-fold increase in cell proliferation. In the study by Navaei et al,^116^ the incorporation of gold nanorods into cardiac patches significantly enhanced cell retention, which increased progressively with higher gold nanorod concentrations.

Vascularization within cardiac patches, as demonstrated by Gálvez-Montón et al,^179^ is crucial to integrate with the host myocardium and support the delivery of oxygen and nutrients to the tissue. The maximum size of cardiac patches is typically in the range of 10–60 mm in diameter.^,91,99,107,117,119,157,160,174,196^ The maximum thickness of cardiac patches is around 1–3 mm.^68,144,147,165^ Lakshmanan et al^79^ sought to address vascularization by incorporating vascular endothelial growth factor and basic fibroblast growth factor into a previously characterized nanofibrous matrix for promoting angiogenesis. The histological examination indicated that the patches exhibited numerous sprouting capillaries and improved myocardial architecture compared with the control groups. Mao et al^54^ used a leaf-venation-directed strategy to enable the organization of cell-hydrogel hybrids into aligned, densely packed structures, which significantly enhances the maturation and functionality of engineered cardiac tissues, as seen in neonatal rat cardiomyocytes. Compared with random cell distributions, these patches demonstrate advanced electrophysiological activity, synchronized contractions, and increased expression of maturation-associated genes.

In electrophysiological analysis, significant variability exists in recorded heart rates across studies, with some cases reporting 30–80 bpm^62,108,163,169,197^ and others reaching up to 100–200 bpm.^69,82,112^ The force-frequency relationship in patches is generally found to be positive up to 2.5–3 Hz, similar to the native human myocardium, which typically plateaus or decreases after 3 Hz.^169^ The action potential duration in the patches (≈280 ms) was comparable with native LV tissue, although smaller patches showed shorter action potential durations.^169^ The conduction velocity in the engineered cardiac patches generally ranged from 35 to 50 cm/s, compared with the 50–60 cm/s typical of native myocardium.^34^

Conductive materials can be used to enhance the functionality of cardiac patches by promoting electrical conductivity, which is vital for the proper synchronization of cardiomyocyte contraction. Common conductive materials used in cardiac patches include polypyrrole (n = 4),^61,76,164,180^ polyaniline (n = 4),^67,97,155,164^ carbon nanotubes (n = 4),^73,80,125,137^ graphene (n = 3),^75,141,175^ gold (n = 10),^43,60,101,115-117,126,135,157,176^ and MXene (n = 2).^74,143^ Importantly, most inorganic conductive materials are nondegradable and are eliminated from the body primarily through excretion. However, their prolonged accumulation could pose potential toxicity risks, underscoring the need for long-term safety evaluations to assess their biocompatibility and ensure their safe use in clinical applications.

Hemodynamic studies assess the modifying effect of cardiac patches on heart function by examining various key parameters, including EF, fractional shortening, and ventricular dimensions at both end-systole and end-diastole as well as infarct size reduction.

Nine small- and large-animal studies^113,117,140,158,164,173,179,184,198^ noted no significant or only slight improvement in EF, which did not reach 50%. Seventeen animal studies reported significant improvements in EF, reaching the normal range of 50%–70%.^61-63,99,140,144,145,147,154,162,197^ Furthermore, 8 animal studies observed a statistically significant reduction in myocardial infarction size.^82,108,117,120,166,167,178,179^ Three preclinical studies reported the largest reduction of 30%–55% of the initial infarct size.^82,166,167^ Although the decrease in infarct size was reported in various ways, such as relative to the remaining ventricular wall versus a reduction compared with the initial infarct size, making direct comparison of percentages challenging. Of 14 large animal studies (13 pigs^60,117,144,145,160,169,170,177-179,185,186,196^ and 1 sheep^139^), 10 studies^60,144,160,169,171,177-179,185,186^ did not report any adverse events. One study^145^ reported perioperative bleeding complications, another study^112^ reported ventricular fibrillation and postoperative infections, and the third study reported anesthetic complications.^196^

A total of 5 clinical studies,^189,191-194^ including 3 reports of single cases,^189,191,192^ investigated the safety and efficacy of various cardiac patches. In 2 case studies, a patch was applied to a nonrevascularizable myocardial scar in a patient who also underwent coronary artery bypass grafting (CABG).^189,191^ Prat-Vidal et al^189^ used a PeriCord (decellularized human pericardium, recellularized with Wharton’s jelly-derived mesenchymal stromal cells) patch. The procedure demonstrated preliminary safety, and the researchers indicated that there was no need for immunosuppression. During the 3-mo follow-up, the patient was alive and without hospital admissions. A 3-mo cardiac MRI revealed a ~9% reduction in scar mass, a 10% reduction in LV end diastolic volume, and a 9% reduction in LV end systolic volume. Bhatt et al^191^documented the use of a second-generation, Food and Drug Administration–approved CorMatrix-ECM patch in a CABG patient. EF increased linearly with 39% in the 5th month, 45% in the 10th month, and 51% in the 14th month. Myocardial fibrosis and scar steadily improved. Clearly, in both studies, it is impossible to distinguish between the effect of the patch and the revascularization surgery.

A third case study^192^ reported the implantation of allogeneic human-iPS cell-derived cardiomyocyte patches (3.3 × 10^7^ cells/patch) in a patient with severe ischemic cardiomyopathy and HF. The patches were implanted via thoracotomy onto the LV epicardium. The patient received immunosuppressant drugs for the first 3 mo postimplantation. Analysis of myocardial displacement using cardiac CT indicated significantly enhanced regional myocardial motion in the transplantation area at 6 mo and 1 y, suggesting substantial angiogenic benefits mediated by cytokine paracrine effects. Furthermore, coronary flow reserve across the myocardium increased from 2.16 to 5.30, with all segmental regions of the LV showing significant enhancement.

Bayes-Genis et al^194^ conducted a phase I, double-blind, single-center clinical trial to evaluate the safety of PeriCord (n = 12 patients, 7 treated and 5 controls). Patients received PeriCord implantation in addition to CABG surgery. In the treatment group, 4 serious adverse events occurred in 2 patients, including respiratory failure and wound dehiscence, but these were expected not to be linked to the PeriCord implant. The PeriCord group exhibited a nonsignificant decrease in infarct size from 10.0% to 8.0%, whereas the control group showed a tendency toward an increase from 9.0% to 10.0%. Due to its first-in-human design and small sample size, the researchers did not aim to detect changes in cardiac function or scar size.

In a first-in-human observational cohort study by Svystonyuk et al,^193^ Cormatrix patches were applied in 8 patients undergoing CABG. Researchers did not observe any device-related complications or adverse events in all 8 patients; however, only 2 patients showed significant results. Cardiac MRI identified 2 patients with improvements in structural (eg, 11 and 13 g reduction in scar mass) and functional metrics 6 mo postimplantation. In these 2 patients, the regions treated with patches and surgical revascularization showed significantly enhanced tissue perfusion relative to areas treated with CABG alone. Global myocardial perfusion also improved consistently over time for both patients. Other patients were not discussed regarding these parameters.

### Hybrid VADs

Hybrid VADs are devices that combine mechanical and biological components to support heart function in patients with HF.^199^ They are designed to enhance biocompatibility and reduce complications, such as thromboembolism and infection, that are commonly associated with traditional mechanical support systems.^200,201^ One of the ongoing challenges associated with VADs is the elevated risk of infection at the driveline exit site. Out of 10 articles reviewed^199,202-210^ in this section, 9 articles^199,203-210^ focused on developing linings to reduce infection rates at the driveline exit site. Because the focus of our review is on the development of BAH and achieving clinically relevant heart function, we determined that studies addressing the biological components of driveline exit site fall outside the scope and chose not to discuss them further in this review.

Ferrari et al^202^ developed a pulsatile displacement pump featuring a hyperelastic membrane that cyclically propels blood similarly to the first-generation VADs. The luminal surface of the membrane is designed with a honeycomb hexagonal topography to support the growth of a living endothelial layer. The hybrid membrane VAD was implanted in 4 sheep, assessing endothelialization in different actuation schemes. The successful development of a mature, growth-arrested endothelial monolayer on the synthetic substrate, coated with cross-linked gelatin and preseeded with adult ovine endothelial cells before exposure to blood at a high density of 10^5^ cells/cm^2^, was confirmed by immunofluorescence after 3 d. This study highlights the ability of the endothelial cells to effectively integrate into the synthetic environment while maintaining their functional properties in vitro.

The hybrid VAD concept is still in its early stages, requiring significant advancements to address challenges in circulatory mechanical support. Although initial efforts have centered on reducing infection rates at driveline exit sites through the incorporation of biological components, achieving full biocompatibility across all device elements is critical for broader clinical success. This entails enhancing hemocompatibility to reduce risks of thrombosis and haemolysis, mitigating immune responses to prevent inflammation and fibrosis, and ensuring mechanical compatibility to minimize tissue damage and promote integration.

### Reporting Quality

The quality assessment for animal studies is reported in Table 3. The analysis of animal studies demonstrates robust reporting in areas such as species description (100%), strain identification (92%), and ethical review approval (83%). However, significant gaps remain in critical details, including the reporting of animal age (34%), weight (41%), and adverse events (9%). Although follow-up duration was clearly specified in 83% of studies, only 45% adequately described random group allocation and blinded echocardiographic assessment. These findings underscore the need for improved adherence to reporting standards, particularly in study design transparency and the documentation of adverse events, to enhance reproducibility and reliability in preclinical research. Quality assessment for clinical studies was not performed, as 3 of 5 clinical studies were case reports.

## DISCUSSION

This systematic review aims to give an overview of new advancements in BAH, engineered myocardium, and hybrid VAD development, evaluating outcomes, as well as their feasibility for clinical translation. The initial literature search yielded 1564 articles, which were screened by title and abstract. The majority of studies were excluded because of the absence of a biological component, lack of relevance to the intervention of interest, or focus on non-HF patients. For instance, studies focusing on the repair of cardiac defects in congenital heart disease or those centered on valvular reconstruction were excluded.

Three hundred four articles remained for full-text screening, with additional 20 publications retrieved from the manual references search through reference chaining. Ultimately, we included 181 studies. The majority of publications (n = 151; 80%) were in the field of cardiac patches. This is reflected in Figure 3, which also illustrates that cardiac patches reached the clinical stage, whereas BAHs are still in the earlier stages of development, primarily in in vitro testing.

### BAH

In BAH development, decellularized tissue is considered a promising biomaterial for heart regeneration. In fact, it retains organ complexity and structure at the macro and microscales, ideally containing biologically active molecules that support cell phenotype and function and preserving its vascularization.^7^ Different chemical agents have been used for decellularization although none of the methods was able to remove all DNA. Indeed, inflammatory reactions and thrombus formation were observed in most BAH studies. After decellularization, the BAH needs to be repopulated by cells. Recellularization efforts have shown some promise, with studies reporting >50% recellularization primarily around injection sites. Although the use of bioreactors has improved cell maturation and integration, the challenge of achieving uniform cell distribution and functionality across the scaffold persists.

Other read-out parameters in BAH research concern electrical and hemodynamic performance. All studies reported only marginal electrical activity. Additionally, the beating frequency of BAH falls short compared with a healthy adult heart. These discrepancies emphasize the need for improved electrical integration to achieve better functionality. In the majority of the studies, assessment of the hemodynamic performance of a fabricated BAH was not reported, most probably because there was no observable contraction. If included, mechanical performance lagged far behind that of native hearts. For instance, the first complete BAH fabrication achieved only about 25% of the neonatal human heart systolic pressure (~15 mm Hg) in a preload and afterload-regulated Langendorff set-up, illustrating the challenge of replicating the contractile force necessary for effective heart function.^6^ The maximum intraventricular pressure of 0.75 mm Hg recorded by Yasui et al^15^ using a catheter measurement is markedly lower and fails to align with native physiological pressures, which typically range from 80 to 120 mmcHg.^34^

These results underscore that the development of BAHs that have the potential to offer an alternative to heart transplantation is still far away. The inability of current BAHs to show any meaningful electrical or hemodynamic performance suggests a need for enhanced scaffold design and recellularization strategies that can better support vascularization, cardiac cell metabolism, and myocardial contractile function.

BAHs offer another potential for advancing cardiovascular research, beyond its clinical applications. By replicating key structural and functional aspects of the human heart, BAHs enable the study of disease mechanisms, such as myocardial fibrosis or HF, in a biologically relevant context. BAHs can also provide a platform for evaluating the efficacy and safety of new therapies, including gene editing, pharmacological agents, and cell-based interventions, under dynamic physiological conditions, offering insights into personalized therapeutic strategies.

### Cardiac Constructs

Results from studies on cardiac constructs highlight ongoing challenges in replicating the architecture and function of natural cardiac tissue. Ninety percent^8-13,36,37^ of the studies focused on in vitro experiments, and there is no clinical study yet.

Similar to the development of BAHs, decellularization/recellularization approaches benefit from the preexisting architecture and vascularization of the ECM, which closely mimics native tissue. However, achieving an adequate cell load remains a major challenge, as engineered constructs currently reach only 8% of the cell density found in native tissue.^37^

The biological assembly approach, although advantageous for integrating a diverse array of cells, is hindered by insufficient vascularization. This limitation is crucial, as it directly affects the muscle construct’s capacity to receive essential nutrients and oxygen, compromising the maturation and viability of the construct.^35^ Another main limitation of this technique is the difficulty in creating 3D complex architecture resembling that of the human myocardium.

Most recent studies rely on 3D bioprinting, which allows for a patient-tailored fabrication of cardiac tissue with structural fidelity as it offers the potential to create complex tissue architectures ranging from cardiac muscle,^35-37^ to entire ventricular construct,^8-13^ and small tubular heart pumps.^38-40^ However, it faces challenges similar to those faced by self-assembly and decellularization/recellularization techniques regarding vascularization and cellular viability. Most studies focus on contractile strength and electrical activity,^9-13,38-40^ yet these metrics remain far from replicating the function of native myocardium.

In addition to this, it is noteworthy that all the ventricle constructs developed and analyzed in this review were limited to dimensions of a few centimeters, with no larger models reported. This raises questions regarding the scalability of these techniques and their applicability to adult-sized ventricular constructs.

Therefore, developing BAHs or ventricles with clinically relevant electrophysiological performance and/or contractile power remains a significant challenge. Integrating biological components with supplementary systems appears essential to achieving the desired beating functionality. In this regard, soft robotics might be explored as an alternative promising method for generating sufficient beating/contractile strength. The use of soft and flexible materials could potentially address the limitations of current rigid devices, which propel blood in a nonphysiological manner. Some research groups focus on soft robotic sleeves around the failing heart,^211^ others focus on soft robotic total artificial heart development.^212^ Combining this with a biological inner layer, like the one that the hybrid heart project envisions,^213^ might further reduce the biocompatibility issues.

### Cardiac Patches

A diverse range of materials has been used in cardiac patch fabrication, ranging from natural to synthetic and composite formulations. Composite materials have emerged as they combine the biological functionality of natural substrates with the superior mechanical properties of synthetic formulations.

Cardiac patches show promise in terms of baseline electrophysiological functions, but many still fall short of achieving the synchronization and conduction velocity of native myocardial tissue. Studies such as Wang et al^198^ and Querdel et al^169^ showed that conduction speeds and voltage outputs remain inferior to those of native myocardium, which remains a crucial barrier to functional integration, as suboptimal electrical properties can increase arrhythmogenic risk.^169,198^

Although improvements in cell proliferation and viability are seen with porous scaffolds,^131^ cell retention and distribution remain inconsistent across different types of scaffolds and cell types. Fabricated patches enhanced with conductive materials, such as carbon or gold, or enriched with vascular endothelial growth factor, have shown better cell adhesion and retention.^79,146^ Without adequate vascular support, the patches may become necrotic, especially if larger, thicker scaffolds (>2 mm) are required to cover significant portions of the myocardium. Therefore, the lack of effective vascularization is a critical limiting factor. Studies by Khorramirouz et al^112^ and Perea-Gil et al^141^ suggested that angiogenesis within patches supports cell survival but only to a limited depth.

Although animal studies show promising results, it is important to emphasize that in more than half of the studies, the outcome assessor (eg, echocardiographist) was not blinded. The predominance of in vitro and preclinical in vivo studies emphasizes the need for clinical trials to further evaluate the effect of cardiac patches in patients. Five clinical studies^189-193^ assessed different patches. As stated before, especially in patients in whom a patch was combined with CABG, it is impossible to differentiate whether an increase in EF, a decrease in LV dimensions, or a reduction in scar mass is due to the revascularization or the patch. In addition, it is impossible to assess whether any observed positive effect on hemodynamic performance is related to the mechanical support of the patch itself (a cap over an infarcted area), contraction of the patch, or to the assumed induced promotion of endogenous tissue regeneration/repair and vasculogenesis, the so-called paracrine effect.

To date, researchers have been unable to develop a myocardial patch capable of replicating the synchronous contractions of native myocardium. Although some patches have mechanical properties that mimic healthy myocardium, it seems very unlikely that applying such a patch onto infarcted myocardium would result in significant improvement in EF or in a reduction in scar mass. Thus, it seems most plausible that the effect of cardiac patches is due to their paracrine effect. Importantly, from the very little clinical experience thus far, we cannot conclude that cardiac patches have any beneficial effect, nor that they are safe.

### Biohybrid VADs

Biohybrid VADs are designed to enhance immunological acceptance and reduce complications, including thrombogenic risks and immune rejection, through the integration of mechanical and biological components. We could find only 1 biohybrid VAD study, the hybrid membrane VAD. The development of a mature, growth-arrested endothelial monolayer on a synthetic substrate, coated with cross-linked gelatin and preseeded with 10^5^ cells/cm², was successfully confirmed by immunofluorescence after 3 d.^202^

## LIMITATIONS

This systematic review has several limitations. First, while it provides a comprehensive overview of state-of-the-art developments in BAHs, VADs, and engineered myocardium, this breadth restricts in-depth analysis of individual topics. Consequently, the review may not capture all relevant publications, potentially leaving gaps in understanding specific niche areas within these subfields. Nevertheless, this breadth allows the review to highlight how various advancements can be synergistically used to drive the field toward successful clinical translation and improve patient outcomes in HF.

Furthermore, the inclusion of a diverse range of study designs—from in silico models to clinical trials—limits the possibilities for direct comparisons of publication quality. In contrast, this heterogeneity can inform the design of future preclinical experimental studies and clinical trials in this field.

## CONCLUSIONS

Overall, progress has been made in the fabrication of BAHs, cardiac constructs, cardiac patches, and biohybrid VADs. Yet, none of the techniques fully replicates the complexity of native cardiac myocardium. More importantly, thus far, outcomes with regard to hemodynamic performance of BAHs or constructs are marginal at best. Cardiac patches show promising results in preclinical studies, with the paracrine effect of the patches being the most plausible explanation of these results. Importantly, however, from the very little clinical experience thus far, it is not possible to definitively conclude that cardiac patches offer any beneficial effects or that they are safe.

All in all, the path toward developing a fully functional BAH or even pieces of functional myocardium appears to be long, extremely complex, and perhaps even unattainable. Combining biological strategies (such as developing an inner lining derived from the patient’s own cells) with nonbiological ways of propelling blood, like soft robotic technologies, probably represents the most promising pathway to make progress toward clinical translation of these innovations. This synergy could yield a biocompatible hybrid heart capable of replicating the structural, mechanical, and functional parameters of a native heart, ultimately addressing critical gaps in mechanical circulatory support and transplantation. However, this path is also long and complex.

## ACKNOWLEDGMENTS

The authors are grateful to Dr Wichor Bramer, biomedical information specialist at the Erasmus MC Medical Library, for his help with designing the Boolean search strings used in this systematic review.

## Supplementary Material
