# Supplementary material for: Bioartificial Hearts, Assist Devices, and Myocardium: New Developments
Source: Transplantation. 2025 Jun 24;109(11):1692–709. doi: 10.1097/TP.0000000000005435 (PMC12539523; doi:10.1097/TP.0000000000005435)
Supplement: Supplementary file 1 [file tpa-109-1692-s001.pdf]

Supplemental Digital Content. Materials and methods

The review encompasses original research studies that include data from patients with HF, preclinical animal models, as well as *in vitro* and *in silico* studies. The search strategy was developed in collaboration with information specialists at the Erasmus MC Medical Library, incorporating a combination of the MeSH terms and keywords related to the fabrication of bioartificial hearts, cardiac patches and constructs, and (bio)hybrid VADs.

This process was conducted blind to each other's scores to maintain objectivity. Disagreements between the researchers were resolved through discussion and careful examination of the pre-defined inclusion and exclusion criteria, with a third senior researcher (P.F.G.) consulted as needed to reach consensus. We included peer-reviewed articles in the English language, which discussed original research in bioartificial heart fabrication, engineered myocardium (cardiac constructs), cardiac patches, hybrid VADs. We also assessed editorials, perspectives, and expert commentaries.

The inclusion criteria were based on the PICO model, encompassing Patients/Population, Intervention, Comparison, and Outcomes.[20] We included studies involving adult and pediatric patients with HF, as well as relevant pre-clinical research. As such, this review encompasses various study designs, including *in silico* modeling, *in vitro* experiments, *in vivo* animal studies, and clinical trials. Studies that focused on extracorporeal support were excluded.

The same selection criteria applied during the title and abstract screening were utilized for the full-text review phase, with eligible articles advancing to the data collection stage. Two researchers (A.R. and P.C.V.) conducted the screening process independently and blinded to each other's scores to ensure maximum objectivity in the

selection process. Any disagreements were resolved through consensus. Furthermore, the researchers employed a background snowballing method or reference chaining, by reviewing the reference lists of key articles and identifying subsequent studies that cite these foundational papers. The selection criteria applied during the main screening were also utilized for the reference chaining stage, with the exclusion of the restriction on the publication year range. This approach allowed for the inclusion of relevant seminal works published before 2014, ensuring that all significant milestones in the field were captured.

For study characteristics, general information about the study was gathered, including authors, publication title, year, design, number of participants, a summary of the aims and methodologies, animal characteristics (species, sex), clinical characteristics of patients (sex, age, comorbidities), follow-up duration, and scaffold materials. In the second category, we focused on techniques used for (bio)fabrication, such as 3D bioprinting and electrospinning, along with any applicable decellularization methods. In preclinical studies, our primary focus regarding outcome was on hemodynamic performance (e.g., ejection fraction (EF), interventricular pressure), mechanical characterization of the materials (e.g., Young's modulus), electrophysiological parameters (e.g., heart rate and amplitude), as well as histological performance (e.g., cell viability, vascularization, immune response). In clinical studies, the primary endpoints, when provided, included myocardial tissue regeneration and hemodynamic performance.
